# ECG Identification Based on the Gramian Angular Field and Tested with Individuals in Resting and Activity States

**DOI:** 10.3390/s23020937

**Published:** 2023-01-13

**Authors:** Carmen Camara, Pedro Peris-Lopez, Masoumeh Safkhani, Nasour Bagheri

**Affiliations:** 1 Computer Science Department, Carlos III University of Madrid, 28911 Leganés, Spain; 2 Computer Engineering Department, Shahid Rajaee Teacher Training University, Tehran 16788-15811, Iran; 3 Electrical Engineering Department, Shahid Rajaee Teacher Training University, Tehran 16788-15811, Iran; 4 School of Computer Science (SCS), Institute for Research in Fundamental Sciences (IPM), Tehran 16788-15811, Iran

**Keywords:** biometrics, ECG, wearables, gramian angular field, deep learning

## Abstract

In the last decade, biosignals have attracted the attention of many researchers when designing novel biometrics systems. Many of these works use cardiac signals and their representation as electrocardiograms (ECGs). Nowadays, these solutions are even more realistic since we can acquire reliable ECG records by using wearable devices. This paper moves in that direction and proposes a novel approach for an ECG identification system. For that, we transform the ECG recordings into Gramian Angular Field (GAF) images, a time series encoding technique well-known in other domains but not very common with biosignals. Specifically, the time series is transformed using polar coordinates, and then, the cosine sum of the angles is computed for each pair of points. We present a proof-of-concept identification system built on a tuned VGG19 convolutional neural network using this approach. We confirm our proposal’s feasibility through experimentation using two well-known public datasets: MIT-BIH Normal Sinus Rhythm Database (subjects at a resting state) and ECG-GUDB (individuals under four specific activities). In both scenarios, the identification system reaches an accuracy of 91%, and the False Acceptance Rate (FAR) is eight times higher than the False Rejection Rate (FRR).

## 1. Introduction and Related Work

Lately, some researchers have proposed the use of physiological signals for cybersecurity purposes [1]. These signals are helpful in a broad amalgam of applications, ranging from authentication and identification systems [2], through key generation functions [3], to cryptographic primitives [4].

The strength of this type of solution is its universality, as every living person has vital signs (e.g., electroencephalograms or electrocardiograms). Signal acquisition can be made without interfering with the users’ daily lives, guaranteeing collectability and acceptability. The permanence of the signal-based solutions is superior to commonly used systems such as passwords or token-based solutions. These approaches offer high performance (e.g., accuracy), proving the uniqueness of each user signal. Finally, low error rates hinder counterfeiting attacks (resistance to circumvention).

**Figure 1:**
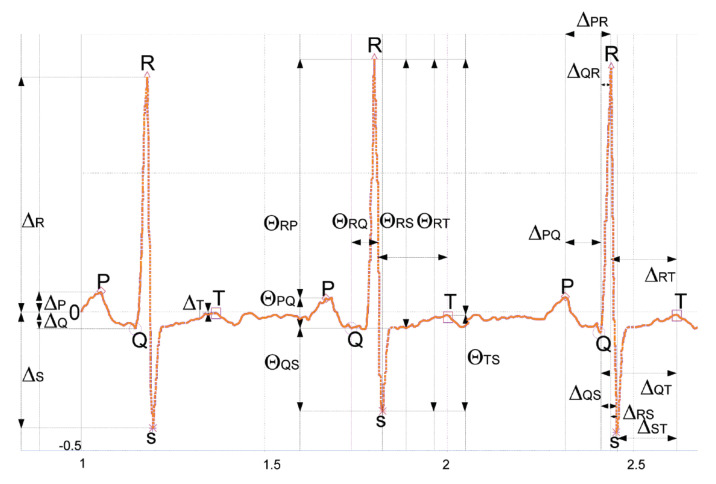
An ECG trace (three beats).

Biopotential signals represent the action potentials originated by a set of different cells [5]. In this work, we focus on electrocardiogram (ECG) records, which represent how the electrical activity of the heart muscle (myocardium) evolves. In particular, changes in the electrical potentials occur due to the contraction (polarization) and relaxation (depolarization) of the myocardium. Five waves make up one cycle of an ECG signal, as depicted in Figure 1. First, the depolarization of the atria occurs (P-wave). Then the Q, R, and S waves (QRS complex) reproduce the ventricle depolarization. Lastly, we have a wave caused by the repolarization of the ventricles (T wave) [6].

Regarding ECG, in the literature, we can find many proposals focused on biometrics identification [7,8,9]. Rathore classified the proposals into two categories: handcrafted and non-handcrafted approaches. Within the former, we can distinguish between fiducials and non-fiducials solutions. The fiducial points of an ECG trace consist, among other things, of amplitude peaks (e.g., R-peaks) or the time intervals between two peaks. (e.g, $\Delta_{RS}$). Using a subset of all possible fiducial points, some authors have built up identification systems [10]. Some authors consider that the extraction of fiducial points may be computationally demanding and propose using statistical features without the necessity of any fiducial point. In this vein, some authors propose to use autocorrelations [11] and others extract features in a transform domain using Discrete Cosine Transforms (DCTs) [12] or Wavelet Transforms (WT) [13].

Nowadays, the trend is a deep learning-based approach [14,15,16], which is within the non-handcrafted category. This ECG identification approach avoids the computation cost of feature extraction while offering proper performance (e.g., high accuracy and low error rate). The first step in many of these approaches is transforming the temporal signal into an image. For instance, spectrograms [17] or spectral correlation images [2] have been tested for that purpose. Alternatively, some authors extract features using a CNN. In this vein, in Ref. [18] the authors proposed to use what they called a “cascade CCN”. As for the two CCNs used, the first one is employed for feature extraction and the second one for classification (user identification).

There has been a remarkable evolution in ECG signal acquisition in recent years. A few years ago, the subject had to visit a cardiologist, who placed seven or nine electrodes on the body to acquire a reliable ECG. Nowadays, due to the proliferation of smartwatches and their advanced functionalities, some of these devices (e.g., Ref. [19] or Ref. [20] already have sensors that can record an ECG trace by touching the device with a finger (i.e., 1-lead ECG). Interestingly, the FDA has accredited some of the newest smartwatches as medical devices [21]. The records collected by these are, therefore, equivalent to those that can be collected in a medical setting. Therefore, wearable devices such as smartwatches or sports bands make biometric systems based on ECG signals more realistic nowadays.

**Contribution:** Cardiologists use electrocardiograms daily in their diagnoses. Apart from that, researchers have also shown the effectiveness of using ECG records for biometrics identification. In this wave, our proof-of-concept proposal is the first contribution—to the best of our knowledge—that proposes to use Gramian Angular Field images to transform the temporal ECG recordings and feed a Convolutional Neuronal Network with the purpose of user identification. We have assessed our proposal using the subject’s recordings from two public and very accepted datasets (MIT-BIH Normal Sinus Rhythm Database [22], and ECG-GUDB [23]) to facilitate the reproducibility of our results. The subjects are at rest in the first dataset, in line with the datasets used in many other previous works. In the second dataset, the subjects were recorded during four activities, and the experiment represents the system’s evaluation under real-world conditions of the subjects’ daily life.

**Organization:** We will explain the paper’s organization shortly. We introduce in Section 2 the used dataset and present the image transformation method used as well as the network used for classification. Then, we interpret the results obtained in our experimentation (see Section 3). Finally, in Section 4, we analyze how our proposed system satisfied the seven properties commonly required by a biometrics system, and we end by extracting some conclusions.

## 2. Methods and Materials

This section starts by explaining the chosen dataset and how we eliminate noise and preprocess each ECG record. After that, we describe how to convert a time series (ECG recording) into an image via the Gramian Angular Field. Finally, we explain the network used for user identification, which is inspired by the VGG19 network.

### 2.1. Dataset and Preprocessing

We can find in the literature many public datasets with ECG recordings [22,24,25]. In most of these datasets, the users suffer a cardiac pathology (e.g., arrhythmias or coronary artery disease) since the recordings were collected in a clinical setting. In our experiments, we employ the MIT-BIH Normal Sinus Rhythm Database (The database is available at https://physionet.org/content/nsrdb/1.0.0/ (last accessed on 1 January 2023).) (Physionet-NSRDB in short) [22] in which cardiologists detected no significant cardiac conditions. Note that the existence of pathologies may introduce a bias in the identification problem we aim to address. The mentioned dataset was collected at Boston’s Beth Israel Hospital and included 13 women aged 20 to 50 and 5 men aged 26 to 45. For each user sample, the recordings of leads ECG1 and ECG2 were acquired. In our experimentation, we use the ECG1 (a modified lead II) inspired by previous works [26,27].

Before working with the ECG recordings, the first step is to clean the signal. The DC component is eliminated first by subtracting the mean value. Next, we cut the noise components (respiration and power-line) using a pass-band filter. Concerning the filter parameters, 0.67 Hz and 0.45 Hz are the lower-cut-off-frequency and the upper-cut-off-frequency used, respectively. All the recordings in the database were cleaned using this process. After this, we split each user recording in windows of *W* seconds. We set the window length to five seconds due to three main reasons: (1) similar values were used in previous works [28]; (2) it includes several heartbeats, and (3) it is a reasonable time window for authenticating a user. Once each ECG record is divided into segments, we convert them into an image using the Gramian Angular Field (GAF), which preserves the temporal dependency. The procedure to compute the GAF is summarized in the following section.

### 2.2. Gramian Angular Field

In 2015, Wang and Oates introduced the concept of the Gramian Angular field to convert a time series into an image. Next, we summarized the math behind this transformation, however, the reader is urged to consult Ref. [29] for all the details. The input is a time series, $X=\{x_{1},x_{2},\cdots ,x_{n}\}$ with *n* observations of real values. First, we rescale the series into the interval $\left[-1,1\right]$:

(1) $${\tilde{x}}^{i}=\frac{(x_{i}-max\left(X\right)+\left(x_{i}-min\left(X\right)\right)}{max(X)-min(X)}$$

Second, we transform the scaled time series into polar coordinates. The timestamp represents the radius and the angular cosine of the time series value as the angle. Mathematically,

(2) $$\left\{\begin{array}{c} \phi =arccos({\tilde{x}}^{i})\\ r=\frac{t_{i}}{N} \end{array}\right.$$

where *N* is a regulation parameter for the polar system.

The above transformation is bijective and preserves the temporal dependency through the *r* coordinate. Finally, we can use the trigonometric sum to represent the temporal correlation between two intervals. This results in the following matrix, which is a quasi-Gramian matrix:

(3) $$\left(\begin{array}{cccc} cos(\phi_{1}+\phi_{1}) & cos(\phi_{1}+\phi_{2}) & \cdots & cos(\phi_{1}+\phi_{n})\\ cos(\phi_{2}+\phi_{1}) & cos(\phi_{2}+\phi_{2}) & \cdots & cos(\phi_{2}+\phi_{n})\\ \vdots & \vdots & \ddots & \vdots \\ cos(\phi_{n}+\phi_{1}) & cos(\phi_{n}+\phi_{2}) & \cdots & cos(\phi_{n}+\phi_{n}) \end{array}\right)$$

**Figure 2:**
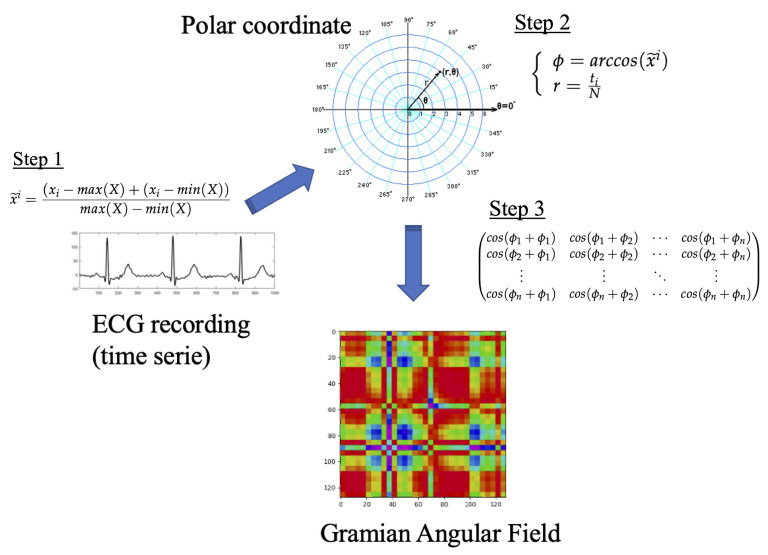
Encoding process of GAF.

Remarkably, we can restore the time series from the values of the main diagonal. The transformation preserves the temporal dependency, and the main drawback is that the resulting matrix is n×n while the input series is 1×n. In Ref. [29], the authors propose to use the Piecewise Aggregation Approximation (PAP) to reduce the size of the matrix. In Figure 2, we outline the transformation process.

### 2.3. Transfer Learning Network

**Figure 3:**
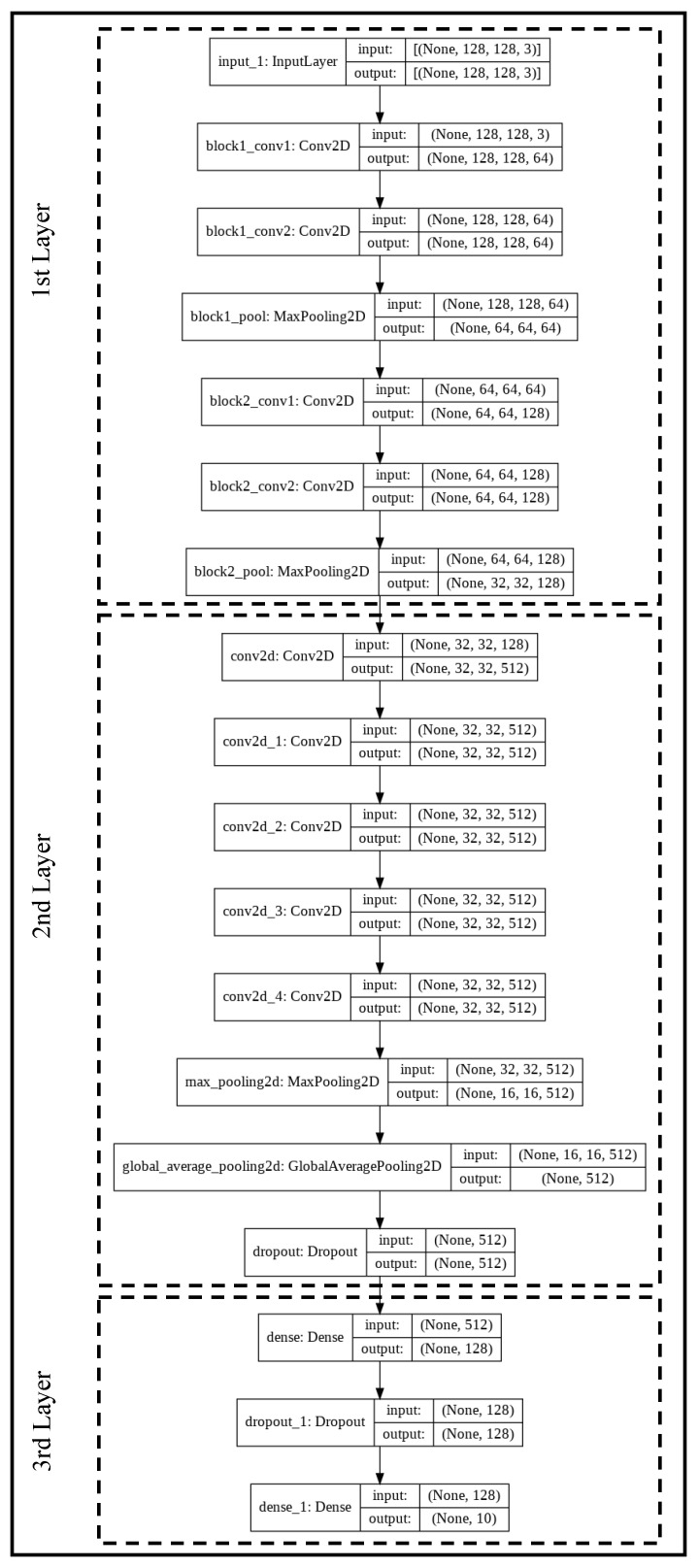
Tuned VGG19 network.

In Figure 3 we sketch the network used for user identification via the Gramian Angular Field (The source code of the network is available at https://lightweightcryptography.com/ECGnetwork.zip (last accessed on 1 January 2023)). The model is inspired by the VGG19 network and uses its first seven layers (see Ref. [30] for details). This first layer of the network aims to extract the relevant features from the input images. For that purpose, apart from the input layer, a block formed by two convolution layers and a max-pooling is repeated two times.

The second layer of the network, which represents its core, consists of five convolution layers (3×3 kernel sizes, ReLU activation, and 512 filters) necessary to complete the features extraction procedure. The number of convolution layers has been tuned to maximize the accuracy and minimize the errors in the final output. After the features extraction, the size of the samples is reduced with two pooling layers: max pooling and global average pooling. Finally, a dropout regularization (25%) to prevent over-fitting ends this layer.

The third network layer is a classification layer (i.e., Fully Connected Layer). It consists of two dense layers to accommodate the output finally to the number (*N*) of existing users (N=10 in our experiments) and a dropout regularization (10%) placed in between. Concerning the dense layers, for the first time, the ReLU is employed as the activation function, and for the second time we opt for the Softmax function to get the probability that a GAF image pertains to a class.

## 3. Results

This section presents our proof-of-concept results when using GAF images extracted from ECG records for identification purposes. To the best of our knowledge, it is the first time the GAF approach has been tested for that particular purpose and analyzed with individuals under resting and activity states. We highlight that our aim is not to provide the best results compared to state-of-the-art (although our results are competitive), but to bring this new and promoting approach to the table.

**Figure 4:**
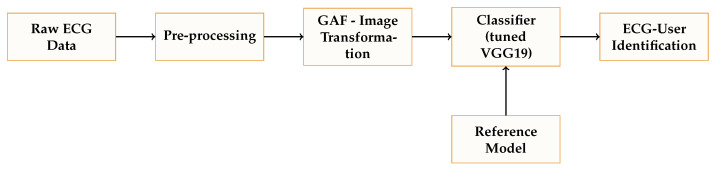
An ECG GAF-based identification system: general structure.

In Figure 4, we summarize the architecture of the proposed identification systems. We have explained each of one the components in the previous section. The classifier is based on the VGG19 network and tests whether an inputted template belongs to one of the legitimate users registered in the system. This approach represents a “one-to-many comparison” system, which, for example, is often used in the access control system of a facility.

We have randomly chosen 10 users from the Physionet-NSRDB dataset in our experiments. For each user, we have selected a sample of 250 min, preprocessed, and divided it into segments (W=5 seconds) as explained in Section 2.1. Then, a GAF image is generated for each segment, producing a set of images for each user (i.e., $N=\frac{250\times 60}{5}=3000$ images). Finally, we divide the total samples (i.e., 3000×10) into training, validation, and test, using a percentage of 80%, 10%, and 10% for each, respectively.

**Figure 5:**
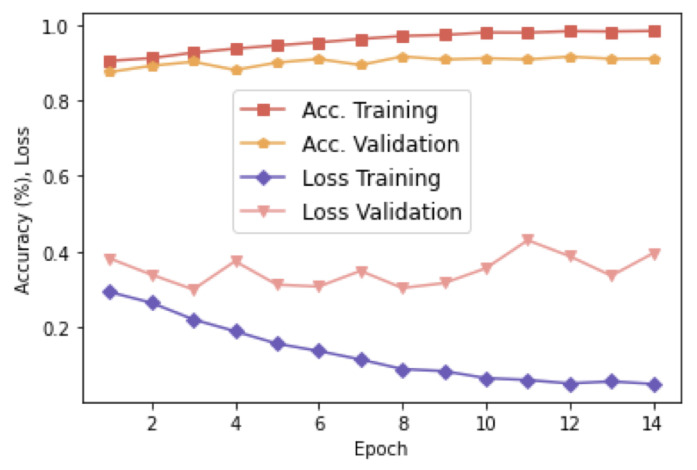
Accuracy and loss: training and validation.

The proposed network (see Figure 3) has around $10^{6}$ trainable parameters that were initialized using the “ImageNet” weights. Besides, we employ the Adam optimizer with a learning rate of $10^{-5}$. We can observe the progression of the accuracy and losses for a different number of epochs in Figure 5. To avoid overfitting, we adopted an early stopping strategy. The validation accuracy is slightly below the training accuracy, but it evolves upwards similarly to the training curve. Concerning losses, we can observe small ups and downs in the validation set, suggesting that it is not necessary to use more epochs.

**Figure 6:**
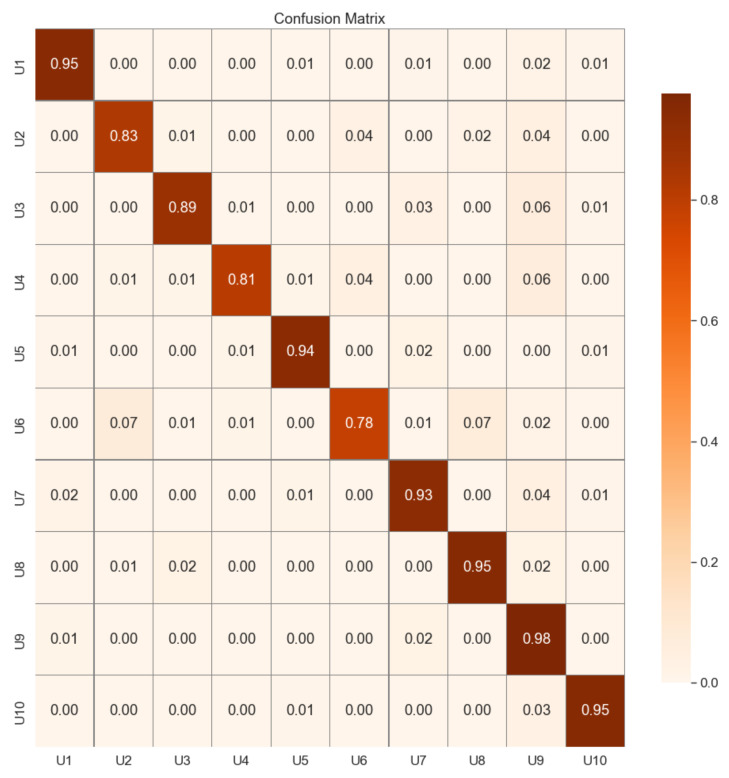
Confusion matrix: validation test.

In Table 1, we summarize the system’s accuracy for the three sets. As it is desirable, the results in validation and testing are equivalents (91%).Regarding the testing dataset, we have analyzed the results in detail (see the confusion matrix in Figure 6). The difference in performance for the 10 subjects is insignificant, and more than half is over 92%, and the misclassification is very low for all the tested users. Users whose accuracy is slightly lower than 90% will have to try twice to authenticate to the system on limited occasions—this is a realistic situation in real scenarios. From these results, we can conclude that the system’s feasibility is guaranteed.

Errors are critical in identification systems, and we use two metrics to assess this issue. The False Acceptance Rate (FAR) represents the percentage of unauthorized users classified wrongly as legitimate. On the flip side, the False Rejection Rate (FRR) is the percentage of valid users mistakenly rejected. Besides, we can calculate the parameter *K* that measures the relation between both metrics (K×FAR=FRR). Note that a value of *K* greater than one means that the unauthorized access is *K* times more costly than locking out a legitimate user. Therefore, a value of *K* greater than one is desirable. In our proof-of-concept, *K* is equal to 9 (FAR=0.01022 and FRR=0.09254). This result is a favorable condition for the system since accepting illegitimate users is the most dangerous condition.

### Analysis with Subjects under a Set of Conditions

One of the main concerns about using biometric systems is how they perform in different situations during our daily lives. In the case of ECG-based biometric systems, we may wonder whether these systems are still effective when the subject’s condition may be affected due to medication or exercise, to mention a few examples. Unfortunately, most of the previous work uses databases acquired in a medical setting where the individuals are only at rest.

To shed light on the behavior of our proposal under different situations, we have analyzed its performance using the ECG-GUDB dataset (The database is available at https://researchdata.gla.ac.uk/716/ (last accessed on 1 January 2023)). In this dataset [23], the ECG signal of 25 users was acquired during 5 activities (sitting, a math test on a tablet, walking on a treadmill, running on a treadmill, using a handbike) for 2 min. The recordings were collected with Attys Bluetooth acquisition device at a sampling rate of 250 Hz and using a standard Einthoven II and III configuration.

The main limitation of this dataset is that the number of samples per subject is not very large—the entire recordings are employed in our experiments for all four activities. We have selected the 10 users for whom we have more samples in our analysis. For each user, once we cleaned the ECG recordings, we split them into segments (W=5 seconds), and a GAF image was obtained for each segment—on average, we got 125 GAF images per user. Then, we split the total samples (125 × 10) into training (80%) and testing (20%).

Utilizing the VGG19 network described in Section 2.3 and with the same configuration parameters used with the Physionet-NSRDB dataset, we have trained and evaluated the model with the samples of the ECG-GUD dataset. The accuracy on training and testing obtained is 95.5 % and 91.6%, respectively. Concerning errors, as is desirable, the False Accepted Rate (FAR=0.0093) is eight and a half times greater than the False Rejection Rate (FRR=0.081). These values are practically identical to those obtained with the Physionet-NSRDB dataset. It implies that GAF images are compelling for subject identification even when the subjects engage in different activities (simulating their day-to-day lives and validating our proposal in real scenarios).

## 4. Analysis and Conclusions

Biometrics systems often demand seven properties: (1) universality; (2) uniqueness; (3) permanence; (4) performance; (5) circumvention; (6) collectability; and (7) acceptability). The previous section shows how our proof-of-concept system achieves high performance while the errors (resistance to circumvention) are low. It is remarkable how the system’s performance remains stable even when subjects are under different activities (i.e., ECG-GUDB dataset). As summarized in Table 2, the vast majority of existing works have used datasets with individuals only at a resting state, which is far removed from a realistic scenario. We have evaluated our solution with two datasets, one with activities, to contend this limitation exiting in previous works. Next, we review the remaining properties.

Our system uses ECG records, which are widely available—we only need to place some electrodes on a body to acquire the signal. Therefore, the used input guarantees the universality property. Besides, the feasibility of using ECG recordings (handcrafted and non-handcrafted approaches) for biometrics identification (uniqueness) has been widely proven in the last years [36]. Our results confirm the feasibility of using ECG signals for building a biometric solution by using a novel approach based on GAF images and a tuned VGG19 classifier.

Concerning permanence, the heart signals are stable over time, although it suffers changes moderately after long periods (i.e., more than five years as explained in Ref. [28]). We can claim that the permanence of ECG records is sufficient and even less demanding in updating terms than the well-known password-based solutions [37].

Other critical parameters are collectability and acceptability. We can argue that these properties are satisfied. On the one hand, nowadays, smart devices (e.g., smartwatches or sports bands) are widely available and accepted in the population. On the other hand, these sorts of devices (e.g., Apple watch [19,38] or Withings Move ECG [20]) can record clear ECG traces, which are even validated for medical purposes. Therefore, collectability and acceptability are satisfied by using the mentioned devices.

Concerning a comparison with state-of-the-art, we emphasize again that our main objective was not to get the best results but to bring to the table the use of GAF functions as a useful transformation for building an ECG-based identification system. In Table 2 we compare our proposal with few representative works; the reader, for instance, can review Ref. [39] for extensive comparatives. Before starting the comparison, we emphasize that the authors only analyze the solutions with subjects in a resting state in practically all existing works. Only in some solutions, such as Ref. [1], the users are in ambulatory condition, but unfortunately, no information is provided about the users’ activities. Regarding the handcrafted solutions, in Ref. [31], or Ref. [32], the authors propose a system based on fiducial points. Although these proposals outperform our solution, the main drawback is the computational cost linked with the extraction of fiducial points. Pinto et al. [12] proposed a non-fiducial-based approach extracting features in a transform domain (e.g., Haar transform), and their results are similar to the ones presented in this article. Another example based on non-fiducial points is the work presented in Ref. [33], which, although outperforming our results, provides no information about the errors, and the reproducibility of the results is not guaranteed due to the use of a proprietary dataset. The last four proposals in the table, similarly to our proposal, are based on deep learning. In Ref. [1], Labati et al. present perfect results in terms of accuracy, but the inputs used to the CCN require the extraction of fiducial points and are twice the length of our input, limiting its usability. The results in Ref. [2], although slightly higher, are comparable to our proposal and, similarly to our work, use a short ECG trace of a few seconds. Interestingly, Zhang et al. presented a multiresolution CNN that slightly surpasses our solution in terms of accuracy, but no values about errors (FAR and FRR) are provided. Finally, Hammad et al. proposed an exciting proposal with high performance and low error rates. Unlikely, the authors obtained their results with datasets with a tiny number of samples per user (e.g., two samples/user in the CYBHi database).

From the above, we can conclude that using Gramian Angular Field images and deep learning is an exciting approach to build novel ECG-based identification systems. In a cybersecurity context and, more precisely, in a biometrics identification context, our proposal is the first work that proposes this approach, to the best of our knowledge, and scrutinizes the solution by using users at a resting state and under different activities (including exercise). Apart from being effective for identification, GAF images allow the recovery of the original ECG record. Due to that, in future work, we will study a system in which the subjects are identified, and at the same time, cardiac ailments (e.g., atrial fibrillation or tachycardia) are detected. Furthermore, phonocardiograms (PPG) signals could be an alternative to ECG records. We chose electrocardiogram records since these signals are much more fruitful in terms of information than the PPGs. The usage combined of both vital signs can also be an interesting future research line.

## Figures and Tables

**Table 1:** Accuracy of the system.

Acc. Training	Acc. Validation	Acc. Test
98%	91%	91%

**Table 2:** A comparative analysis of ECG-based identification solutions.

Proposal	Approach	Database	Accuracy	FRR	FAR
Choi et al. [31]	MLP (handcrafted; fiducial)	Proprietary ${}^{†}$	93.8%	0.085	0.085
Liu et al. [32]	RF (handcrafted; fiducial)	Proprietary ${}^{†}$	93.1%	0.046	0.010
Pinto et al. [12]	MLP (handcrafted; non-fiducial)	Proprietary ${}^{†}$	92.4%	0.033	0.033
Pathoumvanh et al. [33]	ED (handcrafted; non-fiducial)	Proprietary ${}^{†}$	97.0 %	—	—
Labati et al. [1]	CNN (handcrafted; fiducial)	E-HOL-03-0202-003 ${}^{‡}$, PTB ${}^{†}$	100%	0.02	0.02
Abdeldayem et al. [2]	2D-CNN (non-handcrafted; non-fiducial)	CEBSDB ${}^{†}$, Physionet-NSRDB ${}^{†}$ FANTASIA ${}^{†}$ and others	95.6%	0.001	0.022
Zhang et al. [34]	1D-CNN (non-handcrafted; non-fiducial)	CEBSDB ${}^{†}$, Physionet-NSRDB ${}^{†}$ FANTASIA ${}^{†}$ and others	93.5%	—	—
Hammad et al. [35]	CNN VGG-Net (non-handcrafted; non-fiducial)	MWM-HIT ${}^{†}$, PTB ${}^{†}$ and CYBHi ${}^{†}$	96.8%	0.03	0.03
**Our proposal**	Tuned VGG19-net (non-handcrafted; non-fiducial)	Physionet-NSRDB ${}^{†}$	91.0%	0.092	0.0102
		ECG-GUDB ${}^{⊛}$	91.6%	0.081	0.0094

^†^ Subjects at resting state. ^‡^ Subjects under ambulatory recordings (no information about its activities). ^✺^ Subjects under four specific activities (including exercise).

## Data Availability

The databases are available at: https://physionet.org/content/nsrdb/1.0.0/ (accessed on 1 January 2023) and https://researchdata.gla.ac.uk/716/ (accessed on 1 January 2023).

## References

[1] Donida Labati R., Muñoz E., Piuri V., Sassi R., Scotti F. (2019). Deep-ECG: Convolutional Neural Networks for ECG biometric recognition. Pattern Recognit. Lett..

[2] Abdeldayem S.S., Bourlai T. (2020). A Novel Approach for ECG-Based Human Identification Using Spectral Correlation and Deep Learning. IEEE Trans. Biom. Behav. Identity Sci..

[3] Karimian N., Tehranipoor M., Woodard D., Forte D. (2019). Unlock Your Heart: Next Generation Biometric in Resource-Constrained Healthcare Systems and IoT. IEEE Access.

[4] Bai T., Lin J., Li G., Wang H., Ran P., Li Z., Li D., Pang Y., Wu W., Jeon G. (2019). A lightweight method of data encryption in BANs using electrocardiogram signal. Future Gener. Comput. Syst..

[5] Buchner T. (2019). On the physical nature of biopotentials, their propagation and measurement. Phys. A Stat. Mech. Its Appl..

[6] Berkaya S.K., Uysal A.K., Gunal E.S., Ergin S., Gunal S., Gulmezoglu M.B. (2018). A survey on ECG analysis. Biomed. Signal Process. Control..

[7] Li M., Si Y., Yang W., Yu Y. (2022). ET-UMAP integration feature for ECG biometrics using Stacking. Biomed. Signal Process. Control..

[8] Li R., Yang G., Wang K., Huang Y., Yuan F., Yin Y. (2020). Robust ECG biometrics using GNMF and sparse representation. Pattern Recognit. Lett..

[9] Xu J., Yang G., Wang K., Huang Y., Liu H., Yin Y. (2020). Structural sparse representation with class-specific dictionary for ECG biometric recognition. Pattern Recognit. Lett..

[10] Bak E., Choi G., Pan S.B. (2020). ECG-Based Human Identification System by Temporal-Amplitude Combined Feature Vectors. IEEE Access.

[11] Hejazi M., Al-Haddad S., Singh Y.P., Hashim S.J., Abdul Aziz A.F. (2016). ECG biometric authentication based on non-fiducial approach using kernel methods. Digit. Signal Process..

[12] Pinto J.R., Cardoso J.S., Lourenço A., Carreiras C. (2017). Towards a Continuous Biometric System Based on ECG Signals Acquired on the Steering Wheel. Sensors.

[13] Huang Y., Yang G., Wang K., Yin Y. (2021). Multi-view discriminant analysis with sample diversity for ECG biometric recognition. Pattern Recognit. Lett..

[14] Sepahvand M., Abdali-Mohammadi F. (2021). A novel multi-lead ECG personal recognition based on signals functional and structural dependencies using time-frequency representation and evolutionary morphological CNN. Biomed. Signal Process. Control.

[15] Zhang Y., Zhao Z., Deng Y., Zhang X., Zhang Y. (2021). Human identification driven by deep CNN and transfer learning based on multiview feature representations of ECG. Biomed. Signal Process. Control.

[16] Hammad M., Zhang S., Wang K. (2019). A novel two-dimensional ECG feature extraction and classification algorithm based on convolution neural network for human authentication. Future Gener. Comput. Syst..

[17] da Silva Luz E.J., Moreira G.J.P., Oliveira L.S., Schwartz W.R., Menotti D. (2018). Learning Deep Off-the-Person Heart Biometrics Representations. IEEE Trans. Inf. Forensics Secur..

[18] Li Y., Pang Y., Wang K., Li X. (2020). Toward improving ECG biometric identification using cascaded convolutional neural networks. Neurocomputing.

[19] Ringwald M., Crich A., Beysard N. (2020). Smart watch recording of ventricular tachycardia: Case study. Am. J. Emerg. Med..

[20] Maille B., Wilkin M., Million M., Rességuier N., Franceschi F., Koutbi-Franceschi L., Hourdain J., Martinez E., Zabern M., Gardella C. (2021). Smartwatch Electrocardiogram and Artificial Intelligence for Assessing Cardiac-Rhythm Safety of Drug Therapy in the COVID-19 Pandemic. The QT-logs study. Int. J. Cardiol..

[21] Bayoumy K., Gaber M., Elshafeey A., Mhaimeed O., Dineen E.H., Marvel F.A., Martin S.S., Muse E.D., Turakhia M.P., Tarakji K.G. (2021). Smart wearable devices in cardiovascular care: Where we are and how to move forward. Nat. Rev. Cardiol..

[22] Goldberger A., Amaral L., Glass L., Hausdorff J., Ivanov P., Mark R., Mietus J., Moody G., Peng C., Stanley H. (2000). PhysioBank, PhysioToolkit, and PhysioNet: Components of a new research resource for complex physiologic signals. Circulation.

[23] Howell L., Porr B. (2018). High Precision ECG Database with Annotated R Peaks, Recorded and Filmed under Realistic Conditions. https://researchdata.gla.ac.uk/716/.

[24] Srivastva R., Singh A., Singh Y.N. (2021). PlexNet: A fast and robust ECG biometric system for human recognition. Inf. Sci..

[25] Wagner P., Strodthoff N., Bousseljot R., Kreiseler D., Lunze F., Samek W., Schaeffter T. (2020). PTB-XL, a large publicly available electrocardiography dataset. Sci. Data.

[26] Dar M.N., Akram M.U., Shaukat A., Khan M.A. ECG Based Biometric Identification for Population with Normal and Cardiac Anomalies Using Hybrid HRV and DWT Features. Proceedings of the 2015 5th International Conference on IT Convergence and Security (ICITCS).

[27] Lee W., Chang W.W., Jiang X., Chen G., Ishii C., Capi G. (2016). Compressed domain ECG biometric with two-lead features. Proceedings of the First International Workshop on Pattern Recognition.

[28] Camara C., Peris-Lopez P., Tapiador J.E. (2015). Human Identification Using Compressed ECG Signals. J. Med. Syst..

[29] Wang Z., Oates T. Imaging Time-Series to Improve Classification and Imputation. Proceedings of the 24th International Conference on Artificial Intelligence.

[30] Simonyan K., Zisserman A. Very Deep Convolutional Networks for Large-Scale Image Recognition. Proceedings of the International Conference on Learning Representations.

[31] Choi H., Lee B., Yoon S. (2016). Biometric Authentication Using Noisy Electrocardiograms Acquired by Mobile Sensors. IEEE Access.

[32] Liu J., Yin L., He C., Wen B., Hong X., Li Y. (2018). A Multiscale Autoregressive Model-Based Electrocardiogram Identification Method. IEEE Access.

[33] Pathoumvanh S., Airphaiboon S., Hamamoto K. (2014). Robustness study of ECG biometric identification in heart rate variability conditions. IEEJ Trans. Electr. Electron. Eng..

[34] Zhang Q., Zhou D., Zeng X. (2017). HeartID: A Multiresolution Convolutional Neural Network for ECG-Based Biometric Human Identification in Smart Health Applications. IEEE Access.

[35] Hammad M., Liu Y., Wang K. (2019). Multimodal Biometric Authentication Systems Using Convolution Neural Network Based on Different Level Fusion of ECG and Fingerprint. IEEE Access.

[36] Rathore A.S., Li Z., Zhu W., Jin Z., Xu W. (2020). A Survey on Heart Biometrics. ACM Comput. Surv..

[37] Furnell S. (2019). Password meters: Inaccurate advice offered inconsistently?. Comput. Fraud. Secur..

[38] Frisch D.R. (2019). A Novel Technique to Expand the Electrocardiographic Recording Capability from an Apple Watch. Am. J. Med..

[39] Ingale M., Cordeiro R., Thentu S., Park Y., Karimian N. (2020). ECG Biometric Authentication: A Comparative Analysis. IEEE Access.

